# Evidence Suggesting Absence of Mitochondrial DNA Methylation

**DOI:** 10.3389/fgene.2017.00166

**Published:** 2017-11-01

**Authors:** Mie Mechta, Lars R. Ingerslev, Odile Fabre, Martin Picard, Romain Barrès

**Affiliations:** ^1^Section of Integrative Physiology, Novo Nordisk Foundation Center for Basic Metabolic Research, Faculty of Health and Medical Sciences, University of Copenhagen, Copenhagen, Denmark; ^2^Department of Psychiatry and Neurology, Division of Behavioral Medicine, Merritt Center, Columbia Translational Neuroscience Initiative, Columbia University Medical Center, New York, NY, United States

**Keywords:** epigenetics, DNA methylation, mitochondria, bisulfite sequencing, whole genome bisulfite sequencing

## Abstract

Methylation of nuclear genes encoding mitochondrial proteins participates in the regulation of mitochondria function. The existence of cytosine methylation in the mitochondrial genome is debated. To investigate whether mitochondrial DNA (mtDNA) is methylated, we used both targeted- and whole mitochondrial genome bisulfite sequencing in cell lines and muscle tissue from mouse and human origin. While unconverted cytosines were detected in some portion of the mitochondrial genome, their abundance was inversely associated to the sequencing depth, indicating that sequencing analysis can bias the estimation of mtDNA methylation levels. In intact mtDNA, few cytosines remained 100% unconverted. However, removal of supercoiled structures of mtDNA with the restriction enzyme *BamHI* prior to bisulfite sequencing decreased cytosine unconversion rate to <1.5% at all the investigated regions: D-loop, tRNA-F+12S, 16S, ND5 and CYTB, suggesting that mtDNA supercoiled structure blocks the access to bisulfite conversion. Here, we identified an artifact of mtDNA bisulfite sequencing that can lead to an overestimation of mtDNA methylation levels. Our study supports that cytosine methylation is virtually absent in mtDNA.

## Introduction

DNA methylation is a major epigenetic modification which participates in the regulation of gene expression by controlling the access of the transcription machinery to promoters. Previously, we and others have reported that the master regulator of mitochondrial function Peroxisome Proliferator-Activated Receptor Gamma Coactivator 1-alpha (PPARGC1A) can be epigenetically reprogrammed by dietary lipids (Staiger et al., 2005; Rönn et al., 2008; Barrès et al., 2009). While investigating the possible contribution of mitochondrial DNA (mtDNA) methylation in the control of mitochondrial function (and the potential role of dietary factors) would be of great interest, it requires first to clarify on the contentious existence of DNA methylation in the mitochondrial genome.

A seminal study done in mouse and hamster cells showed that mtDNA is readily methylated, although at lower levels compared to nuclear DNA (Nass, 1973). Using assays for quantifying methylation relying on methyl-sensitive restriction enzymes, several groups estimated, in both human and murine cells, that 2–5% of cytosines are methylated in mtDNA (Shmookler Reis and Goldstein, 1983; Pollack et al., 1984). Using methylated DNA immunoprecipitation (MeDIP) to capture mtDNA enriched for 5-methylcytosine or 5-hydroxymethylcytosine followed by real time PCR, apparent methylation of mtDNA was detected in cells from human buffy coat (leucocytes) as well as in different human and mouse cells lines (HCT1116, HeLa, 143B.TK^-^, human fibroblast, MEFs, and 3T3-L1) and piglets (Shock et al., 2011; Bellizzi et al., 2013; Jia et al., 2013, 2015). Moreover, substantial levels of DNA methylation were detected from purified mitochondrial fractions by both ELISA and mass spectrometry (Infantino et al., 2011; Chen et al., 2012; Dzitoyeva et al., 2012; Menga et al., 2017).

Another approach used to quantify DNA methylation is bisulfite sequencing. This assay relies first on the chemical conversion of unmethylated cytosines into uracils and then thymines, whereas methylated cytosines remain intact. Using bisulfite sequencing in human buffy coat cells, cord blood cells, cancer cell lines, or placental tissue (Byun et al., 2013, 2016; Janssen et al., 2015; Zheng et al., 2015; Bianchessi et al., 2016; van der Wijst et al., 2017), as well as mouse liver, brain, or testes (Wong et al., 2013), mtDNA methylation in the D-loop region and in 12S or 16S genes was estimated in the range of 1–20%. Intriguingly, despite the plethora of studies reporting methylation of cytosine exists in mtDNA, several studies using bisulfite sequencing have challenged the presence of methylation in mtDNA by reporting that mtDNA is either not methylated (Dawid, 1974; Hong et al., 2013) or methylated at very low levels (below 2%) (Liu et al., 2016).

In the present study, we aimed to use both targeted- and genome wide bisulfite sequencing approaches to examine the existence of cytosine methylation in mtDNA from mouse and human cells. Our data reveal bisulfite sequencing analysis bias as a possible source of overestimation of mtDNA methylation levels and provides evidence that the secondary structure of mtDNA is a potential source of false bisulfite sequencing positives.

## Materials and Methods

### Cell Culture

Human ovarian cancer cells SKOV3 were a gift from Kristian Helin’s lab (Copenhagen). Human embryonic kidney cells HEK293 and SKOV3 cells were maintained in DMEM with 20% FBS and 1% penicillin-streptomycin in an atmosphere of 5% CO_2_ at 37°C.

Lonza human skeletal muscle myoblasts (Lonza) were maintained in DMEM/F-12, GlutaMAX^TM^ (Gibco, Life Technologies) supplemented with 20% Fetal Bovine Serum (FBS, Sigma-Aldrich) and 1% penicillin-streptomycin (Invitrogen) in 5% CO_2_ at 37°C. At 70–80% confluency, differentiation was induced by reducing the FBS concentration to 2%. Experiments were conducted when cells were fully differentiated myotubes, after 5–7 days in differentiation medium.

### Animals

Wild type C57BL/6J male mice (Taconic Biosciences, Denmark) were 6–8 weeks of age at delivery and single-housed for acclimatization 1 week prior to the start of the experiment. All experiments were conducted in accordance with national Danish guidelines (amendment #1306 of November 23, 2007) as approved by the Danish Animal Experiments Inspectorate (#2014-15-2934-01,027). Mouse muscle tissues were collected from donor mice of the previously published experiment (Jensen et al., 2016).

### Mitochondrial Isolation and mtDNA Extraction

Mitochondria from human myotubes were isolated with an adapted protocol described elsewhere (Mercer et al., 2011). Cells were trypsinized with Trypsin-2.5% EDTA and resuspended in PBS. Cells were resuspended in swelling buffer (10 mM NaCl, 1.5 mM MgCl_2_ and 10 mM Tris-HCl, pH 7.5 all from Sigma-Aldrich) and were allowed to swell for 5 min on ice. Cells were then homogenized seven times with a 7-ml glass homogenizer and sucrose concentration was adjusted to 250 mM. Nuclei were sedimented by centrifugation at 1300 *g* for 3 min at 4°C. The supernatant containing the mitochondria was transferred to another tube and centrifuged at 15,000 *g* for 10 min at 4°C. The mitochondrial pellet was centrifuged for an additional 15 min at 15,000 *g* at 4°C. The mitochondrial pellet was resuspended in PBS and mtDNA was extracted using DNeasy Blood and Tissue Kit (Qiagen) and quantified using Qubit dsDNA HS Kit (Life Technologies).

Mitochondria from mouse *gastrocnemius* were isolated using an adapted protocol described elsewhere (Lanza and Nair, 2009). The muscle was placed immediately into ice-cold muscle homogenization buffer (100 mM KCl, 50 mM Tris-HCl, 5 mM MgCl_2_, 1.8 mM ATP, and 1 mM EDTA pH 7.2, all from Sigma-Aldrich). Fat and connective tissues were removed, and the muscle was minced. Muscle pieces incubated for 2 min in 1 mL of protease medium [1 mL homogenization buffer and 5.66 mg protease from *Bacillus licheniformis*, 10.6 U/mg (Sigma-Aldrich)], washed twice with 3 mL of homogenization buffer, and transferred to a homogenizer containing 4 mL of homogenization buffer. The muscle was homogenized using a motor-driven homogenizer for 5 min at 150 rpm with slow vertical plunger movements. The homogenate was then centrifuged at 900 *g* for 5 min at 4°C. The supernatant containing the mitochondria was collected, and centrifuged a first time at 900 *g* for 5 min 4°C and a second time at 10,000 *g* for 5 min at 4°C to pellet the mitochondria. The pellet was washed once with homogenization buffer, and centrifuged for 5 min at 9,000 *g* at 4°C. The mitochondrial pellet was finally resuspended in PBS and mtDNA was extracted using DNeasy Blood and Tissue Kit (Qiagen). RNase A (Qiagen) treatment was included in the protocol.

### Restriction Enzyme Treatment

Genomic DNA was either untreated or treated with BamHI restriction enzyme (New England Biolabs, cat # R0136) to obtain circular or linearized mtDNA. BamHI recognize and cuts G/GATCC, which is found only at one site in the human mitochondrial DNA at position 14258. The DNA was treated for 4 h at 37°C according to the recommendations from the manufacturer.

### Bisulfite Conversion and PCR

Genomic DNA was bisulfite converted using EZ DNA methylation-lightning kit (Zymo Research, United States) according to the manufacturer’s instructions. The converted DNA was amplified using bisulfite converted primers designed using the online primer design programs BiSearch (Tusnády et al., 2005; Arányi et al., 2006) or Methprimer (Li and Dahiya, 2002) (**Table 1**). Primers for the D-loop, tRNA-f+12S and ND5 targeted the heavy strand, whereas primers for 16S and CYTB targeted the light strand. Primers were checked for specificity using BiSearch to ensure that only mtDNA was amplified. mtDNA amplification was performed using the HotStar Taq plus DNA polymerase kit (Qiagen) according to the manufacturer’s instructions. Briefly, 100 ng converted DNA, 500 μM sense and anti-sense primers and HotStar Taq polymerase 7.5 units/reaction were mixed in a total volume of 50 μl. The PCR was run with the following conditions: 5 min 95°C (60 s 94°C; 60 s 53–55°C; 60 s 72°C) × 35 cycles; 10 min 72°C. Primers were either used separately or multiplexed. When multiplexing primers 200 ng converted DNA was used and the following cycling conditions: 5 min 95°C (60 s 94°C; 90 s 53–55°C; 90 s 72°C) × 35 cycles; 10 min 72°C. PCR products were subjected to gel electrophoresis on 2% agarose (Lonza) and extracted using QIAquick Gel Extraction Kit (Qiagen).

**Table 1:** mtDNA primer sequences used for targeted bisulfite sequencing.

Primer	Sequence	Location	Amplicon size
D-Loop	FWD: AAATCTATCACCCTATTAAC	6–298	292
	RVSE: GTGGAAATTTTTTGTTATGATGT		
D-Loop	FWD: CATAACAAAAAATTTCCACCAAAC	279–458	179
	RVSE: GGGAAAATAATGTGTTAGTT		
tRNA-F/12S	FWD: TTTATATAACTTACCTCCTC	577–765	188
	RVSE: GTGTTTGATGTTTGTTTTTTTTG		
16S	FWD: AATAAATTTATAGGTTTTTAAATTATTAAAT	2763–2873	110
	RVSE: TAACTAATAAAATCTTAACATATACTACTC		
ND5	FWD: TTCAAATATCTACTCATCTTC	12687–12856	169
	RVSE: ATAGGATTGTTTGAATGGTT		
CYTB	FWD: GGTATTATTTTTTTGTTTGTAATTATAGTA	15091–15243	152
	RVSE: CCTCAAATTCATTAAACTAAATCTATCC		

### Bisulfite Sequencing Library Preparation

Library preparation of bisulfite-converted PCR products was performed using NEBNext Ultra DNA Library Kit for Illumina (New England Biolabs) according to the manufacturer’s instructions. In short, 100 ng of PCR product was end-repaired and ligated to adaptors. During PCR amplification indexes from NEBNext Singleplex Kit (New England Biolabs) were introduced and libraries were purified using AMPure beads (Beckman Coulter). Libraries were subjected to 150 bp paired-end sequencing using an Illumina MiSeq instrument (Illumina). Total number of reads was approximately 15 million.

### Whole Mitochondrial Genome Bisulfite Sequencing (WMGBS)

Mitochondria were isolated and mtDNA was purified as described above. Whole mitochondrial genome bisulfite sequencing (WMGBS) was performed using EpiGnome^TM^ Methyl-Seq Kit (Epicentre, Illumina) according to the manufacturer’s protocol. Mitochondrial DNA was sonicated 2 × 10 min 30 Hz using Bioruptor (Diagenode) and bisulfite converted using EZ DNA Methylation-Lightning kit (Zymo Research). 50 ng converted mtDNA was used for library preparation. Random hexamers were added to converted mtDNA following first strand synthesis. After the addition of tag sequences to the 3′ and 5′ ends, a PCR was performed to amplify libraries for 2nd strand synthesis and to add both adaptor and indexes. Libraries were purified using AMPure beads (Beckman coulter) and quality-controlled on a Bioanalyzer 2100 (Agilent Technologies). Libraries were subjected to 150 bp paired-end sequencing using Illumina MiSeq instrument (Illumina).

### Bioinformatics

Whole mitochondrial genome reads and targeted regions were processed similarly: reads were preprocessed with Trim Galore v0.4.1 & Cutadapt v1.12 (Martin, 2011) with the --paired and --trim1 flags set. Due to biases introduced by the primers (visible in an M-bias plot, data not shown) the first nucleotides of each read were trimmed, whole genome reads had 10 nucleotides removed while reads from targeted regions had 2 removed. Preprocessed reads were aligned to the hg38 or mm10 genome depending on dataset using Bismark v0.16.3 (Krueger and Andrews, 2011) assisted by Bowtie2 v2.2.8 (Langmead and Salzberg, 2012) and with the --dovetail flag set. Whole mitochondrial genome reads were de-duplicated using the Bismark de-duplication module. Reads mapping to the mitochondrial genome were extracted using samtools v1.3.1 (Li et al., 2009). Finally, methylation individual methylation levels were extracted using Bismark methylation with the flags --cytosine_report and --CX_context.

Further analysis and visualization was done in the R statistical environment (R Development Core Team, 2014), using the packages data.table (Anon, 2017) and ggplot2 (Wickham, 2009). Testing for the effect of linearizing mtDNA was done using a sign-test and adjusted by Bonferroni correction. Smoothed conditional means were calculated using the “gam” method and a span of 0.2. All correlations calculated are Pearson correlations.

### Statistical Analysis

Statistical analysis was performed using R statistical environment software. In whole genome bisulfite sequencing correlations between estimated methylation and sequencing depth were based on the ∼6,000 to ∼12,000 mitochondrial cytosines covered by the experiment with an estimated methylation greater than 0. The Pearson correlation coefficient was calculated between the logarithm of the number of reads covering a cytosine and the logarithm of the estimated methylation. A *P*-value ≤ 0.05 was considered statistically significant.

## Results

### Detection of Mitochondrial DNA Methylation Is Inversely Correlated with Sequencing Depth

**FIGURE 1:**
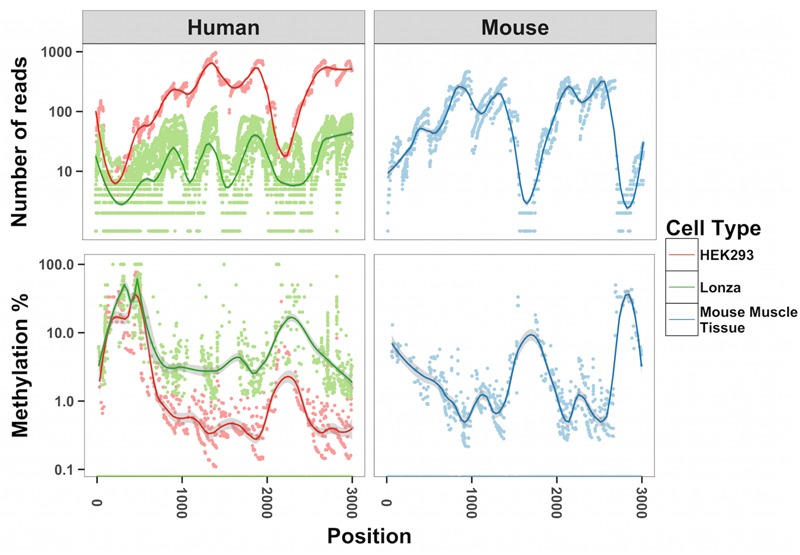
Inverse relationship between methylation of mtDNA and sequencing depth. The number of reads and methylation percentage is shown in the mitochondrial genome from position 1000–3000 on the sense strand. In red, human HEK293 cells; in green, human primary muscle cells; in blue, mouse skeletal muscle. We analyzed all cytosines in each of the samples corresponding to 6,000–12,000 cytosines.

**FIGURE 2:**
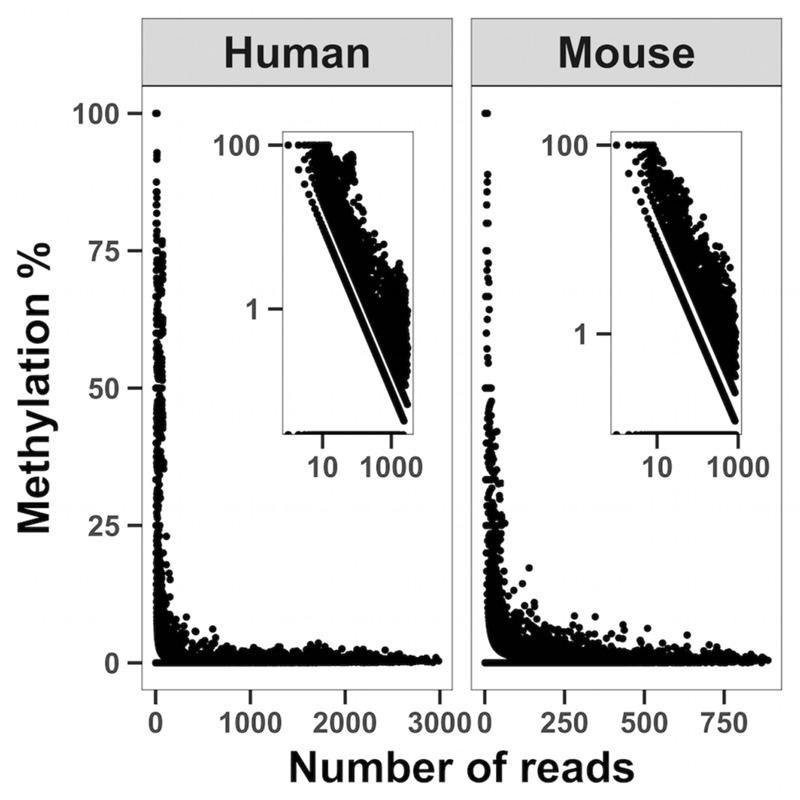
Correlation analysis between sequencing depth and cytosine unconversion rate. WMGBS shows an inverse relationship between mtDNA cytosine unconversion rate (Methylation %) and sequence depth (Number of reads) in both human and mouse cells. Log log scales are shown in the upper right corner. The correlation coefficient is *R* = –0.907, *P* < 2.2E-16 and obtained by pooling all samples.

To map methylation patterns across the whole mitochondrial genome at single base-pair resolution, we performed whole mitochondrial genome bisulfite sequencing (WMGBS). Methylation of mtDNA has been detected in mouse skeletal muscle tissue (Wong et al., 2013) but not in human Epithelial Kidney cells (HEK293) (Hong et al., 2013). Thus, we chose mouse muscle tissue, human muscle and HEK293 cells to study mtDNA methylation. While cytosine unconversion rate in WMGBS was detected up to 100% at certain positions, suggesting 100% methylation at a few specific mtDNA sites, it was striking to observe that cytosine unconversion rate was negatively associated with sequencing depth: in human cell lines and in mouse skeletal muscle, mtDNA methylation at specific cytosine residues was higher when these residues had a low sequencing coverage (**Figure 1** and Supplementary Figures 1A–E). Indeed, calculation of the Pearson correlation coefficient between mtDNA methylation and sequencing depth returned a very tight negative correlation (*R* = -0.907, *P* < 2.2E-16; **Figure 2**). The fact that mtDNA from HEK293 cells had an overall higher sequencing depth than other cells or tissues and was associated to a lower detection of methylation levels, further supports that sequencing depth can influence the estimation of mtDNA methylation (**Figure 1**).

### NUMTs Do Not Cause Overestimation of mtDNA Methylation

Given the inevitable contamination of mtDNA preparation with nuclear DNA (nDNA), nuclear insertions of the mitochondrial genome, named NUclear MiTochondrial segments (NUMTs) (Ramos et al., 2011), represent a potential source of analysis bias after WMGBS. While short NUMTs and NUMTs with low sequence similarity are easy to computationally distinguish from mtDNA, longer NUMTs or NUMTs with sequence similarities could influence the quantification of mtDNA methylation. However, we found no correlation between methylation levels and the length of NUMTs (*R* = 0.06, *P* = 0.11; Supplementary Figure 2A), and only a marginal correlation between methylation and NUMT alignment score, a score established to represent how well each NUMTs align to mtDNA (Ramos et al., 2011) (*R* = -0.1, *P* = 0.006; Supplementary Figure 2B). Bisulfite conversion reduces the sequence complexity of unmethylated DNA and thus, distinguishing NUMTs from mtDNA could be more difficult after bisulfite conversion. To ensure that we could distinguish NUMTs in our analysis, we extracted all possible 300 bp reads from both methylated and unmethylated NUMTs and aligned them back onto the genome using the same setting as in the main analysis. Of the 900.000+ possible reads, none of them aligned to the mitochondrial genome. These results indicate that in our mtDNA preparations, contamination of nDNA was not at the origin of the methylation signal.

### The Secondary Structure of mtDNA as a Source of Overestimation of Methylation Levels

**FIGURE 3:**
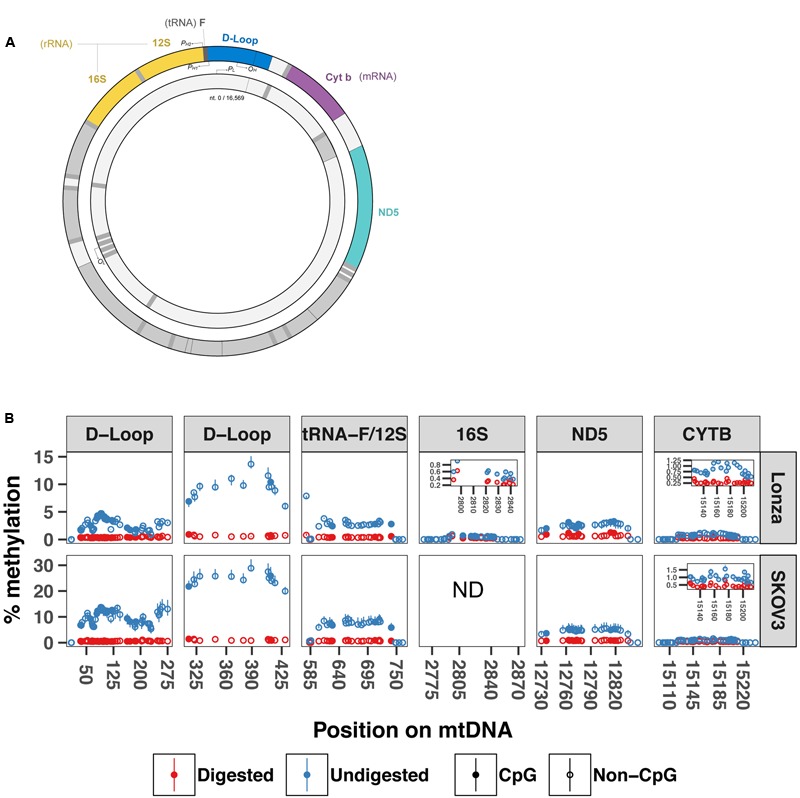
BamHI digestion prior to bisulfite sequencing decreases cytosine unconvertion rate. Targeted bisulfite sequencing was used to compare undigested and digested DNA methylation levels at five different regions of the mtDNA from human muscle cells and SKOV3 cells (*N* = 3). **(A)** Drawing displays the mtDNA regions investigated by targeted bisulfite sequencing. **(B)** Percentage methylation for undigested and digested mtDNA. Full circle represents cytosines in CpG context whereas open circle is cytosines in non-CpG context. Results are presented with a min-max interval and a sign test was used to test for significant methylation differences. D-loop (6–298) *P* = 2.02E-41 (Lonza) and *P* = 1.40E-24 (SKOV3); D-loop (279–458): *P* = 5.00E-12 (Lonza) and *P* = 8.20E-08 (SKOV3); tRNA-F+12S: *P* = 9.22E-15 (Lonza) and *P* = 1.29E-9 (SKOV3); 16S: *P* = 6.50E-05; ND5: *P* = 1.70E-05 (Lonza) and *P* = 9.97E-13 (SKOV3); CYTB: *P* = 2.96E-09 (Lonza), and *P* = 6.68E-13 (SKOV3). D-loop (6–298) includes origin of replication and tRNA-F+12S includes heavy strand promoter 2. P*_H1_*: heavy-strand promoter 1; *P_H2_*: Heavy-strand promote 2; P*_L_*: Light-strand promoter; OH: Origin of replication from heavy-strand; O*_L_*: Origin of replication from light-strand. ND: not determined.

The difference in sequencing depth across the mtDNA that we detected in our WMGBS analysis could originate from the secondary structure of mtDNA. The mtDNA genome can be organized in coiled and supercoiled secondary structures (Kolesar et al., 2013). During sonication of mtDNA, a step of the WGBS protocol, certain mtDNA fragments that are less tightly engaged in the mtDNA supercoiled structure have, in theory, the potential to be preferably freed and ligated to sequencing adapters. This would result in an overrepresentation of mtDNA fragments that do not engage in supercoiling. We tested the hypothesis that mtDNA supercoils can be a source of overestimation of methylation by subjecting DNA to digestion with the *BamHI* restriction enzyme prior to targeted bisulfite sequencing to remove any secondary structure and linearize mtDNA. We focused our analysis on five mitochondrial regions near the origin of replication and transcription start site for the heavy strand: the displacement Loop (D-Loop), tRNA phenylalanine and 12S ribosome (tRNA-F+12S), 16S ribosome (16S), NADH dehydrogenase 5 (ND5) and cytochrome b (CYTB) mRNA-encoding gene (sequencing primers are shown in **Table 1**) in two human cell lines; In human myotubes and the ovarian cancer cell line SKOV3. mtDNA methylation has previously been detected in SKOV3 cells (van der Wijst et al., 2017). In the D-loop (position 6–298), we found methylation levels from 0–4.8% in Lonza and 0–16.9% in SKOV3 cells with undigested mtDNA. In contrast, the same region after *BamHI* digestion showed substantial lower methylation levels (0–0.6%, 0–1.1% *P* = 2.02E-41, *P* = 1.40E-24). Similarly, in the position 279–458 of the D-loop, we detected 5.5–15.1% and 0.5–1% methylation in undigested vs. digested mtDNA from Lonza, respectively, (*P =* 5.00E-12) whereas for SKOV3 cells, we detected 19–32.3% and 0.8–1.5% (*P* = 8.20E-08). In the regions encoding tRNA phenylalanine and the 12S ribosomal RNA and 16S ribosomal RNA, the methylation percentage dropped from 0–7.9% to 0–1.1% (*P =* 9.22E-15) and 0–0.9% to 0–0.6% (*P* = 6.50E-05) in Lonza and 0–11.1% to 0–1.1% in SKOV3 cells (*P* = 1.30E-9), respectively. Finally, in the ND5 and CYTB regions the drop was from 0–3.8% to 0–1.3% (*P* = 1.7E-05) and 0–1.2% to 0–0.5% (*P =* 2.95E-09) after digestion for LONZA and from 0–7.9% to 0–1.1% (*P* = 1.0E-12) and 0–1.8% to 0–0.8% (*P* = 6.68E-13) for SKOV3 cells, respectively (**Figure 3**). These results suggest that mtDNA secondary structure impairs bisulfite conversion and infer that the secondary structure of mtDNA is a source of overestimation of methylation levels in bisulfite sequencing.

## Discussion

Here, we used targeted- and whole mitochondrial genome bisulfite sequencing to determine the methylation profile of mtDNA. Our whole mitochondrial genome approach showed a very tight correlation between sequencing depth and methylation levels at specific cytosines, thereby revealing a potential bisulfite sequencing artifact. Removal of the mtDNA secondary structure by restriction enzyme digestion prior to sequencing dramatically decreased cytosine unconversion rate. Our findings support that cytosine methylation is absent in mtDNA and provide technical insight into the possible source of overestimation of mtDNA methylation levels.

Our whole mitochondrial genome bisulfite sequencing analysis showed that methylation levels were higher for sequences with low coverage, but not with high sequencing depth. This observation indicates that (1) some mtDNA regions were less represented than others in the sequencing libraries and (2) these low-abundant sequences are carrying higher methylation levels. We think that such observation results from the secondary structure of mtDNA. The mtDNA exists in many topological structures in the mitochondria: as open circles, supercoiled, or as catenane circles (linked circles), with the amount of the different topological structures varying between cell and tissue types (Pohjoismäki and Goffart, 2011). The compact, secondary structures of mtDNA are likely to prevent bisulfite conversion, where unmethylated cytosines can escape deamination and stay intact. This would result in false positive methylation signal. In addition, supercoiled structures have been shown to protect the DNA from shear forces (Catanese et al., 2012). Thus, the compact secondary structure of mtDNA may also affect the ability of certain mtDNA regions to be released by sonication during the preparation of WMGBS libraries. Together with blocking access of sodium bisulfite, these structures may decrease the representation of certain mtDNA sequences and explain our finding that cytosine conversion rate is associated with low sequencing coverage. The inverse relationship between sequencing depth and methylation levels disappeared when regions were sequenced at a high depth of 250x coverage. To our knowledge, a relationship between coverage and methylation levels has not been observed in whole genome bisulfite sequencing despite the commonly used sequencing coverage of 10x. This reinforces the notion that mtDNA carries specific properties that justifies adaptation of the bisulfite sequencing protocol when interrogating mtDNA methylation.

After linearizing mtDNA prior to bisulfite conversion, cytosine unconversion rate was abolished. Our hypothesis that the secondary structure of mtDNA leads to bisulfite sequencing artifact is further supported by the high methylation levels we detected in undigested samples on a portion of the D-loop (15.1% for Lonza and 32.3% for SKOV3 cells), where Mitochondrial Transcription Factor A (TFAM) binds and induces a U-turn structure (Ngo et al., 2011). When mtDNA was digested prior to sequencing, methylation at the D-loop region dropped to below 1.5% for both cell lines. These results strongly indicate that the secondary structure of mtDNA is a source of overestimation in bisulfite-based mtDNA methylation analysis. Masking of cytosine by protein-DNA interactions is a cause of bisulfite sequencing artifact, as showed by lower cytosine conversion rate in DNA fractions where protein removal is incomplete (Warnecke et al., 2002). Similarly, the observation that higher denaturation temperatures lead to higher cytosine conversion rates indicates that secondary structures may mask cytosine from bisulfite conversion and lead to an overestimation of methylation levels (Clark et al., 1994). Recently, it was shown that methylation levels are found at lower amounts in circular- compared to linear mtDNA, with methylation levels below 2% in linearized mtDNA (Liu et al., 2016). In the cell types we used for the currently study, methylation levels observed after mtDNA digestion were at most 1.5%. The discrepancy between our results (maximum 1.5%) and the earlier reported (maximum 2%) (Liu et al., 2016) could be caused by cell-type specific DNA methylation levels. However, our previous tests, using spiked-in unmethylated template into the bisulfite reaction, showed that 1.5% methylation signal corresponds to background levels in targeted bisulfite sequencing (Barrès et al., 2009). Thus, this suggests that the level of methylation detected in the mitochondrial genome corresponds to noise background. The consistency of very low levels of linearized mtDNA methylation across different cell types further stresses the importance of restriction enzyme digestion to avoid secondary structure-induced overestimation of bisulfite-based detection of methylation levels. For future studies investigating mtDNA methylation, *BamHI* or *BglII* (Kolesar et al., 2013) may be used for human and mouse cells, respectively, thereby ensuring an appropriate estimation of mtDNA methylation.

Change in human mtDNA methylation has been associated with environmental exposure to pollutants (Janssen et al., 2015; Zheng et al., 2015) and different disease states (Feng et al., 2012; Pirola et al., 2013; Baccarelli and Byun, 2015). By targeted bisulfite sequencing, we detected overall higher methylation levels in cancer cells as compared to human myotubes when DNA was not digested prior to bisulfite conversion. However, after digestion, methylation levels dropped below 1.5% in all cell lines. Thus, our observation supports that the secondary structure of mtDNA, rather than absolute cytosine methylation could be altered by disease state. Further studies, using a systematic digestion approach prior to bisulfite conversion, are warranted to conclude on the association between disease and changes in mtDNA secondary structure.

Using both targeted- and whole genome MeDIP, several reports have shown an enrichment for both 5 mC and 5 hmC in the mitochondrial genome (Shock et al., 2011; Bellizzi et al., 2013; Ghosh et al., 2014, 2016; Jia et al., 2015). Given that methylated capture-based methods like MeDIP measure exclusively the relative, and not the absolute level of DNA methylation, their use for detection of very low levels of methylation may be disputed. Indeed, methylation enrichment might be technically biased by capture efficiency at low methylation levels and lack of appropriate normalizing method or, contamination with NUMTs in sample. Other non-chemical methods, for instance mass spectrometry, could in principle be used to quantify mtDNA methylation however, due to the contamination of nDNA in mtDNA preparations, quantification by mass spectrometry is likely to lead to overestimation of mtDNA methylation levels.

The presence of NUMTs in the nuclear genome makes methylation of certain areas of mtDNA very challenging to assess. For instance, we identified a large region of the mouse mtDNA (position 6390–11042) with 100% identity with genomic DNA. Due to mapping issues, we could not retrieve any methylation data from that particular mtDNA region. In this study, we sequenced reads of 300 bp. In humans, none of the 755 known NUMTs have spans of 300 bp or more with 100% mtDNA identity (Ramos et al., 2011). Thus, sequencing fragments of 300 bp minimizes greatly the risk of contamination of NUMTs in WMGBS. For WMGBS, we only used reads mapping uniquely to the mitochondrial genome and for targeted bisulfite sequencing, we used Bisearch primer design (Tusnády et al., 2005; Arányi et al., 2006) and selected only regions that were completely unique to mtDNA. Designing primers outside NUMTs greatly decreases the risk of false positives.

## Conclusion

We identified a likely source of an artifact as a cause for the overestimation of cytosine methylation in mtDNA and provide technical insight for bisulfite sequencing of mtDNA. A systematic use of restriction enzyme digestion prior to bisulfite conversion, using not only various cell lines, tissues and species but also various pathological and physiological states, is warranted to help conclude on the existence of cytosine methylation in mtDNA.

## Author Contributions

MM, LI, and RB contributed to data analysis. MM, LI, MP, and RB contributed to the study design. MM and OF contributed to data acquisition. MM, LI, OF, MP, and RB contributed to data interpretation and manuscript drafting and approved the final version of the manuscript. RB is the guarantor of this work and, as such, had full access to all the data in the study and takes responsibility for the integrity of the data and the accuracy of the data analysis.

## Conflict of Interest Statement

The authors declare that the research was conducted in the absence of any commercial or financial relationships that could be construed as a potential conflict of interest.
