# Supplementary material for: Evidence Suggesting Absence of Mitochondrial DNA Methylation
Source: Front Genet. 2017 Nov 1;8:166. doi: 10.3389/fgene.2017.00166 (PMC5671948; doi:10.3389/fgene.2017.00166)
Supplement: Supplementary file 1 [file Image_1.pdf]

Supplementary Material

Evidence suggesting absence of mitochondrial DNA methylation

Mie Mechta, Lars Roed Ingerslev, Odile Fabre, Martin Picard, and Romain Barrès*

*** Correspondence:** Corresponding Author: barres@sund.ku.dk

Supplementary Figure 1. Methylation of mtDNA and sequencing depth are inversely correlated in human cells and mouse skeletal muscle. The number of reads and methylation percentage are shown in the mitochondrial genome from position **(A)** 0-3000 antisense **(B)** 4900-6900 sense, **(C)** 4900-6900 antisense **(D)**14000-16000 sense and **(E)** 14000-16000 antisense. In red, human HEK293 cells; in green, human primary muscle cells (Lonza); in blue, mouse skeletal muscle. We analysed all cytosines in each of the samples corresponding to between 6,000-12,000 cytosines.

A

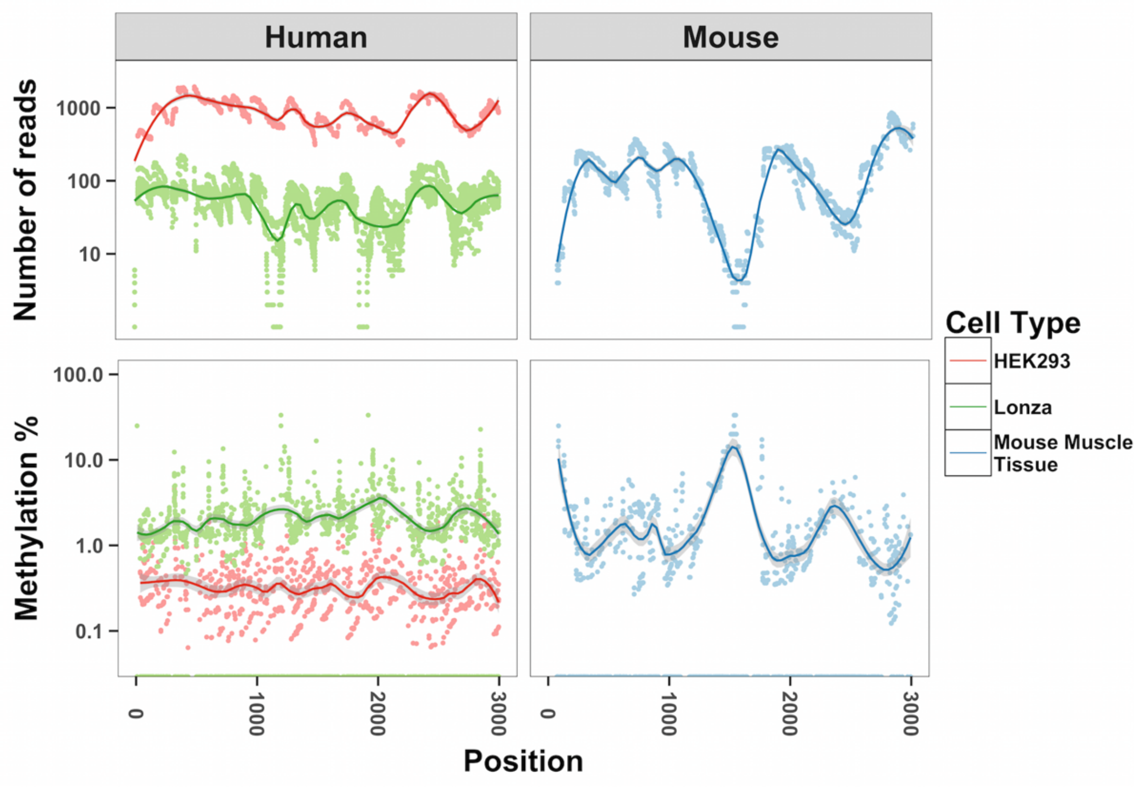

B

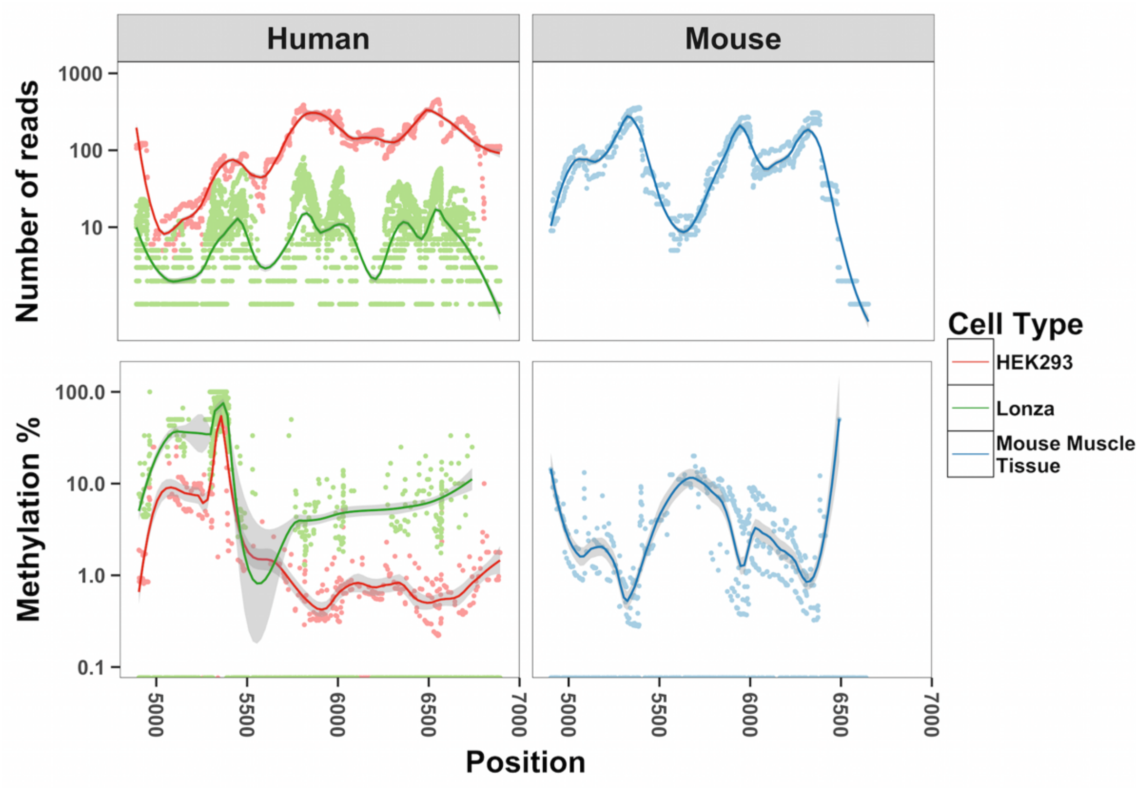
Human
Mouse
Number of reads
1000
100
10
Methylation %
100.0
10.0
1.0
0.1
5000
5500
6000
6500
7000
Position
Cell Type
HEK293
Lonza
Mouse Muscle Tissue

C

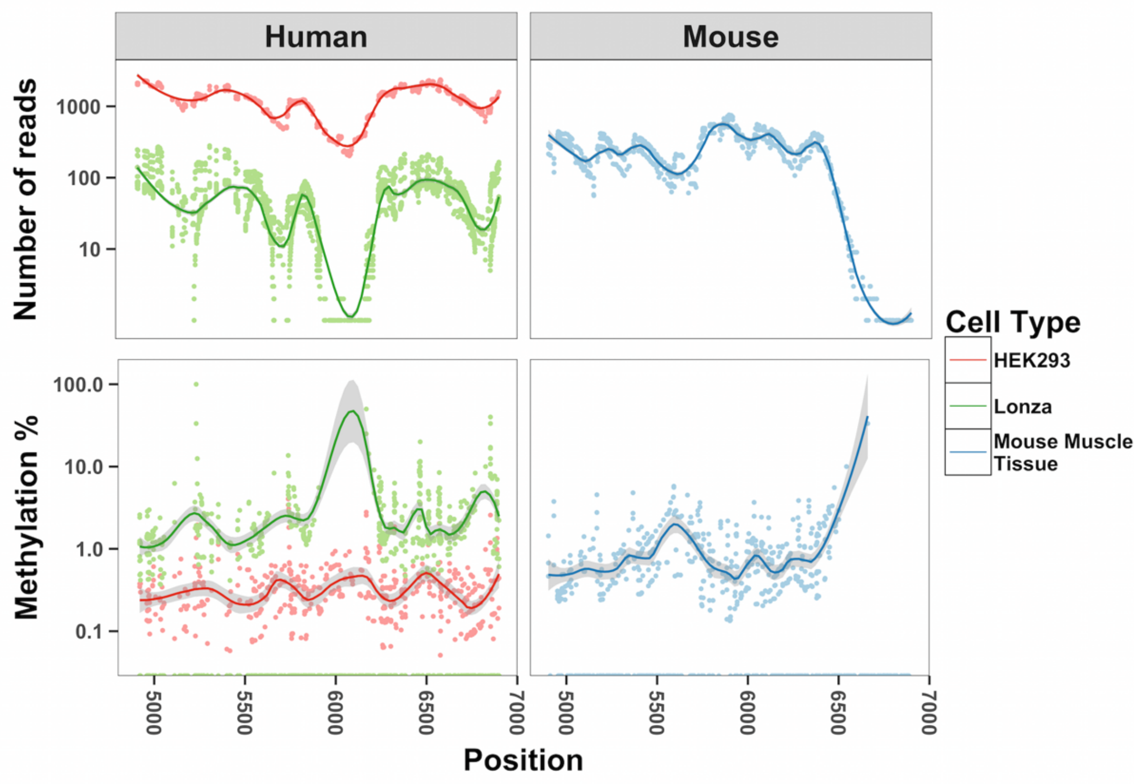

Human
Mouse
Number of reads
1000
100
10
Methylation %
100.0
10.0
1.0
0.1
5000
5500
6000
6500
7000
Position
Cell Type
HEK293
Lonza
Mouse Muscle Tissue

D

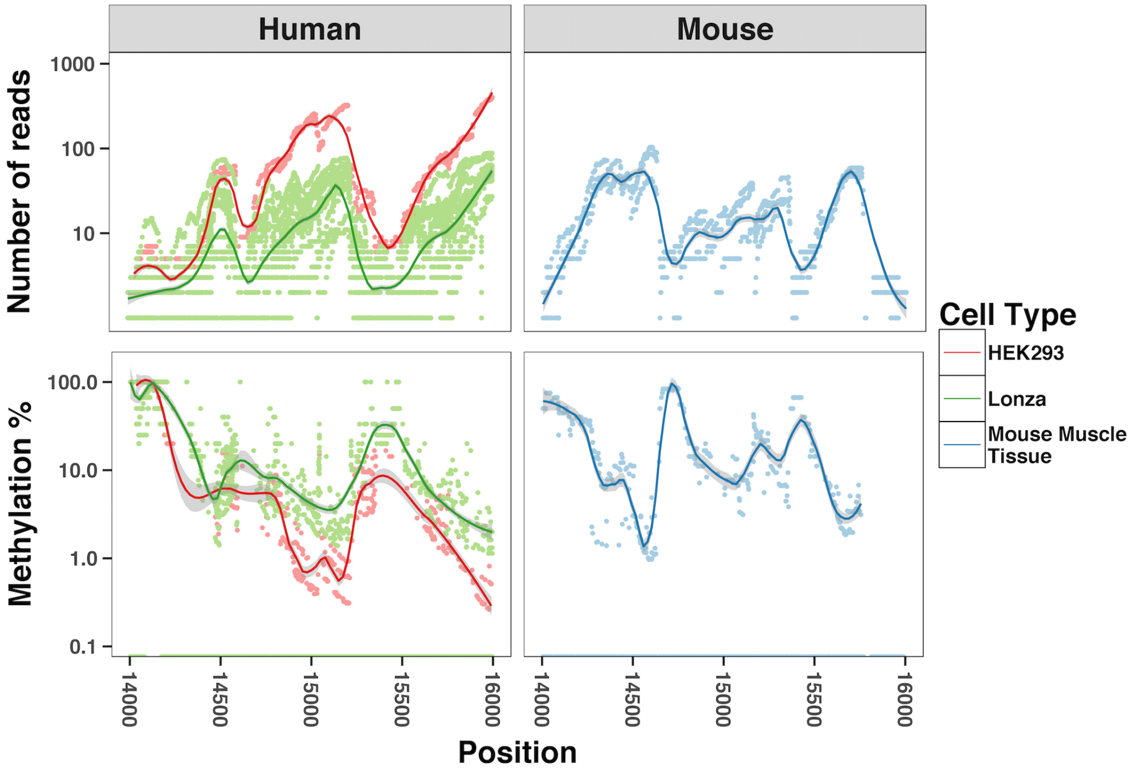

Human
Mouse
Number of reads
1000
100
10
Methylation %
100.0
10.0
1.0
0.1
14000
14500
15000
15500
16000
Position
Cell Type
HEK293
Lonza
Mouse Muscle Tissue

E

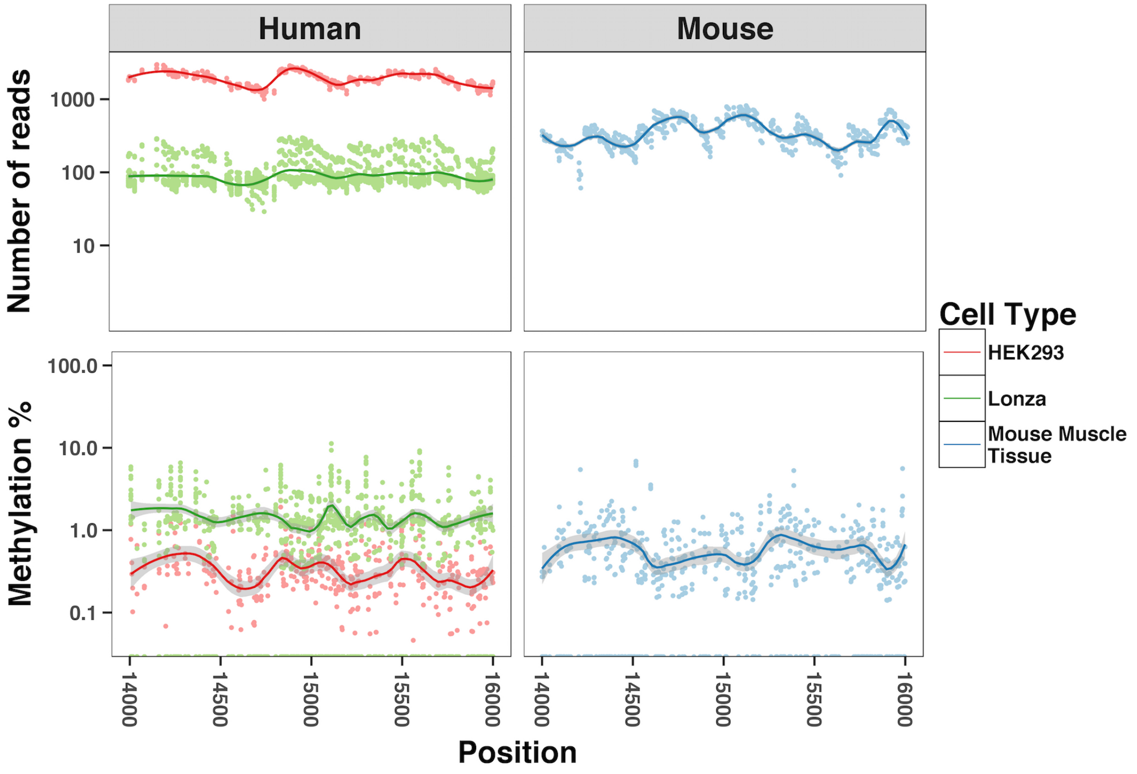

Human
Mouse
Number of reads
1000
100
10
Methylation %
100.0
10.0
1.0
0.1
14000
14500
15000
15500
16000
Position
Cell Type
HEK293
Lonza
Mouse Muscle Tissue

Supplementary Figure 2. Role of NUMTs in mtDNA methylation levels. (A) Correlation between mtDNA methylation and NUMT length (R=0.06, *P*=0.11). **(B)** Correlation between mtDNA methylation and NUMT score (R=-0.1, *P*=0.006). The NUMT score is described elsewhere (Ramos et al. 2011) and is a score representing how well each NUMTs align to mtDNA.

A

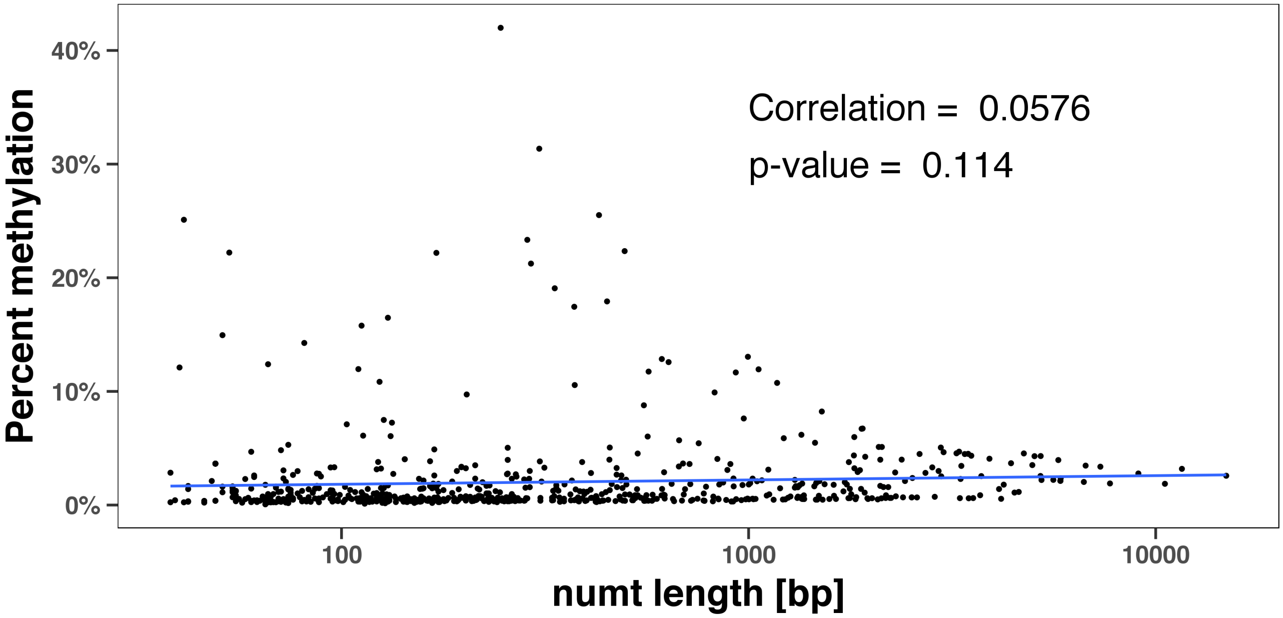

B

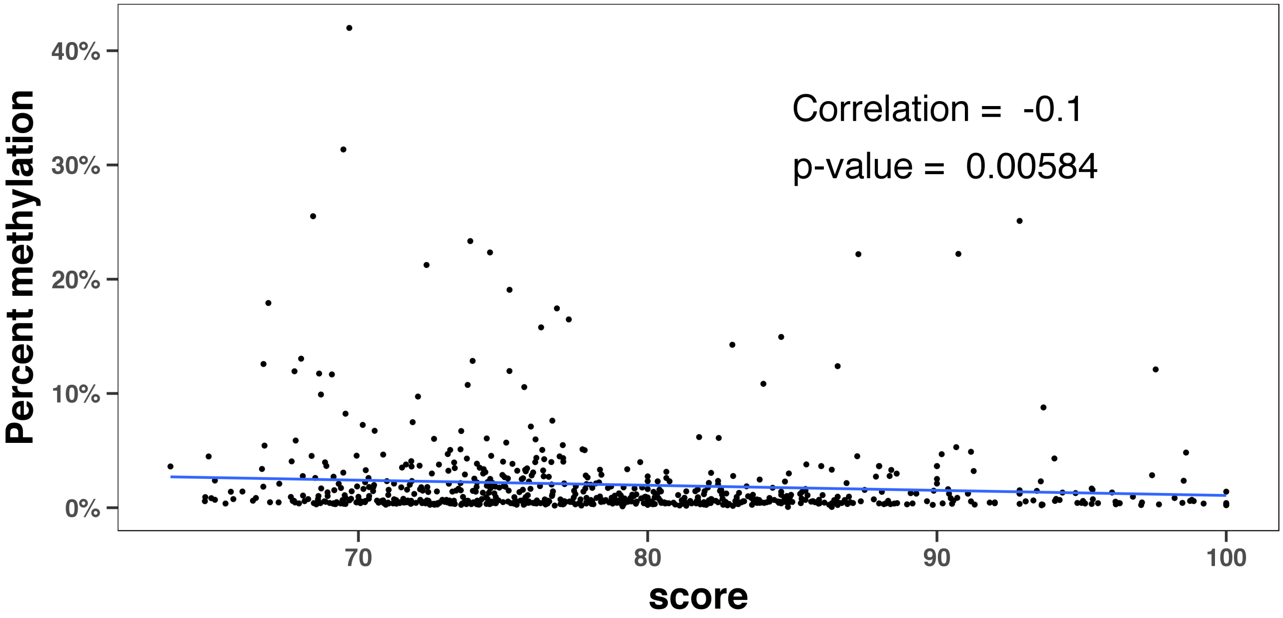

REFERENCE

Ramos, A. et al., 2011. Nuclear insertions of mitochondrial origin: Database updating and usefulness in cancer studies. *Mitochondrion*, 11(6), pp.946–953.
